# The Effects of Antioxidants on the Changes in Volatile Compounds in Heated Welsh Onions (*Allium fistulosum* L.) during Storage

**DOI:** 10.3390/molecules27092674

**Published:** 2022-04-21

**Authors:** Sang Mi Lee, Dami Kim, Young-Suk Kim

**Affiliations:** 1 Department of Food and Nutrition, Inha University, Incheon 22212, Korea; smlee21@inha.ac.kr; 2 Department of Food Science and Biotchnology, Ewha Womans University, Seoul 03760, Korea; kimdami940@naver.com

**Keywords:** Welsh onion, volatile compounds, heat treatment, antioxidant, storage

## Abstract

Welsh onion (*Allium fistulosum* L.) is usually used to enhance the flavor characteristics of various foods. Volatile compounds in Welsh onions, including sulfur-containing compounds, may vary during heat process and storage. Accordingly, the changes in the volatile compounds in Welsh onions, subjected to heat and antioxidant (ascorbic acid and glutathione) treatments during storage, are investigated in the present study. The majority of sulfur-containing compounds in Welsh onions showed significant differences between the untreated Welsh onions and heated Welsh onions. During the heating of the Welsh onions, some sulfur-containing compounds, such as 2-methylthiirane, 1-(methyldisulfanyl)prop-1-ene, 1-[[(E)-prop-1-enyl]disulfanyl]propane, 1-(propyltrisulfanyl)propane, 1-[[(E)-prop-1-enyl]trisulfanyl]propane, and (methyltetrasulfanyl)methane, showed significant differences between the untreated and heated Welsh onions (*p* < 0.05). In addition, partial least square discriminant analysis (PLS-DA) was applied to discriminate the heated Welsh onion samples added with different antioxidants. The heated Welsh onion samples added with ascorbic acid was mainly associated with 2-phenylacetaldehyde, acetic acid, methylsulfanylmethane, prop-2-ene-1-thiol, undecan-2-one, and (2E,4E)-deca-2,4-dienal. Moreover, the key volatile compounds in the heated Welsh onion samples added with glutathione were 3-ethylthiophene, 1-(methyldisulfanyl)-1-methylsulfanylpropane, 1-methylsulfanylpentane, 2-prop-2-enylsulfanylpropane, and 1-propan-2-ylsulfanylbutane.

## 1. Introduction

*Allium* plants are widely used to improve flavor characteristics in several dishes, such as soups and stews, as ingredients and seasoning agents [1]. These *Allium* plants, such as Welsh onion, garlic, and onion, contain various volatile compounds, which can produce a unique taste and flavor [2]. In particular, sulfur-containing compounds in Welsh onions (*Allium fistulosum* L.), such as 1-(methyldisulfanyl)propane, (1E)-1-(methyldisulfanyl)-1-propene, (1E)-1-(propyldisulfanyl)-1-propene, and dipropyltrisulfane, can be produced from several precursors, including 3-[(1E)-1-propen-1-ylsulfinyl]-L-alanine (isoalliin), 3-[(S)-methylsulfinyl]-L-alanine (methiin), and 3-(allylsulfinyl)-L-alanine (alliin), through the enzymatic hydrolysis and non-enzymatic decomposition [3].

Some studies found that these sulfur-containing compounds in the *Allium* genus can be changed by processing methods, such as high-temperature heating, drying, roasting, crushing, and aging [2,3,4,5,6,7,8]. In addition, *Allium* sulfides can be produced by thermal decomposition during the heating process, and their structural changes can occur by thermal reactions [7]. Accordingly, organosulfur compounds in Welsh onions can be changed during processing and storage, leading to the chemical transformation of various volatile components that can significantly affect the flavor quality [9,10].

Several studies have been conducted to improve the shelf life and quality of Welsh onions [11]. The volatile compounds in Welsh onions were affected by oxidizing conditions, which are inevitable in food systems [12]. These oxidizing conditions can be caused by various factors, such as temperature, moisture, gas, and pH levels during processing and storage, which spontaneously create oxidizing agents [13]. Reactive oxygen species and radicals produced under oxidizing conditions react with them, and can then lead to the loss of their original properties, resulting in a deterioration in the quality of food systems [14]. Therefore, antioxidants can be used to maintain the quality properties of Welsh onions during storage.

Ascorbic acid, which prevents browning and improves the nutritional value, can be found in many food systems, either naturally or as an antioxidant additive [15]. In addition, ascorbic acid is an antioxidant that can be classified as a singlet oxygen or oxygen scavenger, and it can react with free radicals to remove it [16]. Glutathione, which is well known as an antioxidant, transforms itself into oxidized glutathione (glutathione disulfide, GSSG), which breaks the disulfide bond of the surrounding substances and reduces it to cysteine [17]. This oxidized glutathione (GSSG) undergoes a cycle that is reduced again by a reductase or chemical reaction [17]. As a non-enzymatic mechanism, glutathione turns into an oxidized form and prevents the oxidation of other substances, or forms disulfide by combining sulfur with itself to prevent oxidation [17].

Various studies have been conducted to investigate the changes in the volatile compounds in the *Allium* plant after the heat process [18,19]. However, to date, studies on the effects of antioxidants on the changes in the volatile compounds in heated Welsh onion have not been conducted. Therefore, the objectives of this study are to investigate the effects of heat treatment and the application of antioxidants on the changes in the volatile compounds in Welsh onions during storage.

## 2. Results and Discussion

### 2.1. The Changes in the Volatile Compounds in Welsh Onions by Heat Treatment

The volatile compounds in untreated and heat-treated Welsh onion samples were analyzed using gas chromatography-mass spectrometry (GC-MS) combined with solid-phase microextraction (SPME). A total of 81 identified volatile compounds in the untreated and heat-treated Welsh onion samples are listed in Table 1. Among these volatile compounds, sulfur-containing compounds, such as 2-methylthiirane (propylene sulfide); 1-(methyldisulfanyl)prop-1-ene; 1-[[I-prop-1-enyl]disulfanyl]propane (propI(E)-1-propenyl disulfide); 1-(propyltrisulfanyl)propane; 1-[[(E)-prop-1-enyl]trisulfanyl]propane; and (methyltetrasulfanyl)methane (dimethyl tetrasulfide), were significantly increased (*p* < 0.05) after the heat treatment.

In addition, some sulfur-containing compounds, such as 5-methylthiophene-2-carbaldehyde, 1-(methyldisulfanyl)propan-2-one (methyl-2-oxo-propyl disulfide), and 1-(methyldisulfanyl)-1-methylsulfanylpropane (methyl 1-(methylthio)propyl disulfide), were only detected in the heated Welsh onion samples. In particular, the levels of 1-(methyldisulfanyl)prop-1-ene; 1-[[(E)-prop-1-enyl]disulfanyl]propane; 1-[[(E)-prop-1-enyl]trisulfanyl]propane; 1-(propyltrisulfanyl)propane; (methyltrisulfanyl)methane; (methyltetrasulfanyl)methane; and 2,5-dimethylthiophene were the most abundant in both the untreated and heated Welsh onion samples, and their levels significantly increased (*p* < 0.05) (except methyltrisulfanyl methane and 2,5-dimethylthiophene) after the heat treatment. It is known that a thermal reaction can affect the formation of sulfur-containing compounds in Welsh onions through their thermal decomposition and rearrangement [19]. Previous studies showed that sulfides are formed in Welsh onion samples when the Welsh onions are exposed to heat during distillation [19,20]. Block et al. [8] also demonstrated that bis(1-propenyl)disulfide, a common compound of *Allium* distilled oil, rearranged to thiophene at a high temperature (85 °C). This compound could then form dimethyl thiophene or dimethyl disulfides [21,22]. Another study reported that cyclic sulfur compounds can be formed through polymerization or degradation at room temperature and polymerization at higher temperature [22], in accordance with the results of our study. In addition, these sulfur-containing compounds, such as (methyltrisulfanyl)methane(dimethyl trisulfide) (sulfurous, cooked, onion, savory, meaty odor notes); 1-(methyldisulfanyl)prop-1-ene(methyl (E)-1-propenyl disulfide) (baked, garlic, onion odor notes); and 1-[[(E)-prop-1-enyl]disulfanyl]propane ((E)-propenyl propyl disulfide) (sulfurous, cooked, onion odor notes) can contribute to the strong odor characteristics of heated Welsh onions [23,24]. This result indicates that these sulfur-containing compounds are considered to be the major contributors to the change in the aroma characteristic of Welsh onions by heat treatment.

Some volatile compounds, such as propanoic acid; heptanoic acid; pent-1-en-3-ol; (E)-hex-2-en-1-ol; tridecan-2-ol; (2E)-3,7-dimethylocta-2,6-dien-1-ol; methyl acetate; methyl propanoate; (2E,4E)-hexa-2,4-dienal; (E)-2-ethylbut-2-enal; (2E,6Z)-nona-2,6-dienal; (E)-non-2-enal; (2E,4E)-nona-2,4-dienal; butane-2,3-dione; 3-(methyltrisulfanyl)prop-1-ene; and 3-methylthiophene-2-carbaldehyde, were only detected in untreated Welsh onion samples. In addition, most of the aldehydes tended to decrease or disappear after heating. It can be considered that those aldehydes underwent thermal decomposition or oxidation during heating [18].

### 2.2. The Effects of Antioxidants on the Change in the Volatile Compounds in Heated Welsh Onions during Storage Periods

The volatile compounds in the heated Welsh onion samples added with antioxidants, such as ascorbic acid and glutathione, during storage periods are listed in Table 2. In total, 65 volatile compounds were identified: 2 acids, 5 alcohols, 16 aldehydes, 1 ester, 2 furans, 5 hydrocarbons, 3 ketones, and 31 sulfur-containing compounds (3 thiols; 7 sulfides; 6 disulfides; 4 trisulfides; 1 tetrasulfide; 8 cyclic polysulfides; and 2 others).

The abundances of most volatile compounds in heated Welsh onion samples tended to decrease according to the storage time. Most of the aldehydes showed such behavior; however, some of them, such as (E)-oct-2-enal and decanal, were not changed significantly in the sample added with glutathione, compared to the other samples during 3–7 days of storage time. In addition, benzaldehyde, (2E,4E)-hepta-2,4-dienal, and (2E,4E)-deca-2,4-dienal were not detected in the samples added with glutathione during the storage periods.

The contents of most sulfur-containing compounds, except for thiols, decreased significantly according to storage periods. The contents of some linear and branched polysulfides, such as 1-(methyldisulfanyl)prop-1-ene, 1-[(E)-prop-1-enyl]disulfanyl]propane, (methyltrisulfanyl)methane, 1-(propyltrisulfanyl)propane, 1-[(E)-prop-1-enyl]trisulfanyl]propane, and (methyltetrasulfanyl)methane, which were the most abundant volatile compounds in the heated Welsh onion samples, decreased with the storage periods. These are known to be the predominant compounds in Welsh onions, and can also be related to the flavor characteristics of *Allium* plants [19,20,21,22,23]. The disulfide bonds of sulfur compounds can be cleaved to produce thio radicals and these radicals are attached to the double bond of another disulfide molecule to form a polysulfide [25]. In addition, the loss of a hydrogen molecule from the alkylthio group can form branched sulfides. In contrast with these sulfur compounds, the contents of some sulfides, such as methylsulfanylmethane (dimethyl sulfide), 3-prop-2-enylsulfanylprop-1-ene (diallyl sulfide), and (methyldisulfanyl)methane (dimethyl disulfide), were relatively low and decreased according to the storage periods. These sulfides can significantly influence the odor characteristics of Welsh onions, due to the low thresholds of such odor notes (unique cooked onion, cooked cabbage, and garlic-like odor notes), ranging from 0.16 to 1.2 ppb [26].

However, cyclic sulfur compounds, such as 2,4-dimethylthiophene, 2,3-dimethylthiophene, 2,5-dimethylthiophene, and 3,4-dimethylthiophene, slightly increased during the initial storage periods (1 and 2 days), possibly due to the rearrangement and cyclization of the fragments from other sulfur-containing compounds. In addition, 3,5-dimethyl-2-(methylthio)-thiophene was increased in the samples added with antioxidants (ascorbic acid and glutathione), according to the storage periods in this study, possibly because highly reactive radicals can attack the linear structure of compounds to form another ring structure [25].

The contents of most sulfur compounds showed a tendency to decrease in the heated Welsh onion samples without antioxidants, according to the storage periods. On the other hand, the thiols and sulfides showed a tendency to increase in the samples added with antioxidants at the initial periods of storage (up to 3 days), whereas they decreased at later periods of storage. Some sulfur compounds, such as 1-methylsulfanylpentane (amyl methyl sulfide) and 1-propan-2-ylsulfanylbutane (butyl isopropyl sulfide), were identified only in the samples added with glutathione, possibly because hydrogen sulfide released from glutathione can participate in the formation of these sulfur compounds by the polymerization and rearrangement of non-enzymatic mechanisms [27]. In addition, it can be assumed that, when a large amount of hydrogen sulfide was produced according to the storage periods, it would react with unstable free radicals to produce other compounds.

**Figure 1:**
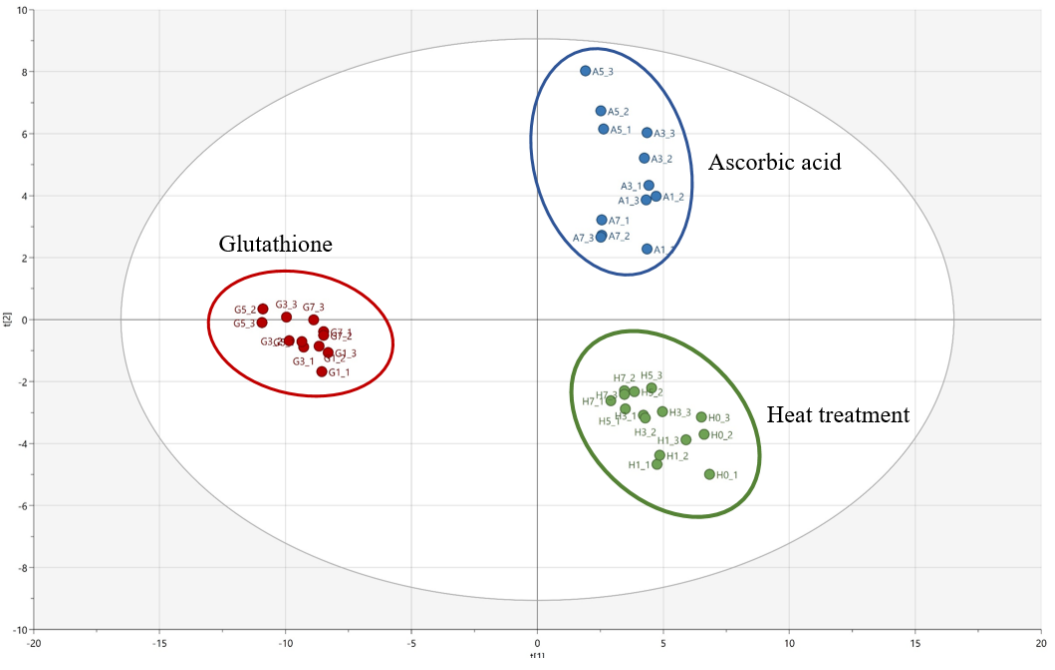
PLS-DA score plot of the volatile compounds identified in the heated Welsh onion samples added with antioxidants during storage.

Partial least square discriminant analysis (PLS-DA) was conducted to investigate the effects of different antioxidants on the change in the volatile compounds in the heated Welsh onion samples during storage. Figure 1 shows the PLS-DA score plot for the comparison of the volatile compounds in heated Welsh onion samples added with antioxidants (ascorbic acid and glutathione). PLS (partial least square) component 1 (PLS 1) and PLS component 2 (PLS 2) explained 38.0% and 26.3% of the variance, respectively, and, hence, together explained 64.3% of the total variance. The parameters of the cross-validation modeling were component 3, R^2^X = 0.49, R^2^Y = 0.93, and Q^2^Y = 0.40. A permutation test involving 100 iterations was also conducted to validate the model, which yielded R^2^ = 0.056 and Q^2^ = −0.282.

The heated Welsh onion samples without antioxidants (control) and heated Welsh onion samples added with ascorbic acid were located on the positive PLS 1 axis, while heated Welsh onion samples added with glutathione were located on the negative PLS 1 axis. In addition, the heated Welsh onion samples added with ascorbic acid were located on the positive PLS 2 axis, whereas heated Welsh onion samples without antioxidants (control) were located on the negative PLS 2 axis. Table 3 and Table 4 list the major volatile compounds identified samples with a criterion of the variable importance plot (VIP) > 1.0.

The negative PLS 1 axis was related to some compounds, such as 3-ethylthiophene; 1-(methyldisulfanyl)-1-methylsulfanylpropane; 1-methylsulfanylpentane; 2-prop-2-enylsulfanylpropane; 1-propan-2-ylsulfanylbutane; 1,3-dithiane; methanethiol; 1-(propyldisulfanyl)propane; propane-1-thiol; (Z)-1-methylsulfanylprop-1-ene; and undecan-2-one, which could be considered as the major contributors to the heated Welsh onion sample added with glutathione (Table 3). On the other hand, 2-phenylacetaldehyde; acetic acid; methylsulfanylmethane; prop-2-ene-1-thiol; undecan-2-one; (2E,4E)-deca-2,4-dienal; 1-[[(E)-prop-1-enyl]trisulfanyl]propane; 2-prop-2-enylsulfanylpropane; 1,3-dithiane; 1-(propyltrisulfanyl)propane; 1-methyl-2-(3,5-dimethylthien-4-yl)disulfide; 3,4-dimethylthiophene; and propane-1-thiol were also associated with the positive PLS 2 axis. These results demonstrate that these compounds could considerably influence the heated Welsh onion samples added with ascorbic acid (Table 4).

## 3. Materials and Methods

### 3.1. Materials

Welsh onions (*Allium fistulosum*) were purchased from Nonghyup (Goyang-si, Gyeonggi-do, Korea). The Welsh onions were cultivated in Jangseong-gun, Jeollanam-do in South Korea (2020). All the samples were stored at 25 °C in a temperature and humid chamber (Han Baek Scientific Co., Bucheon-si, Korea), before they were analyzed. The solid-phase microextraction (SPME) fibers and holders were purchased from Supelco (Bellefonte, PA, USA), whereas the vial and screw caps (Ultraclean 18 mm) were purchased from Agilent Technologies (Santa Clara, CA, USA). β-damascone was purchased from Sigma-Aldrich (St. Louis, MO, USA). The mineral oil was purchased from Sigma-Aldrich (St. Louis, MO, USA). The methanol and water were purchased from J.T.Baker (Phillipsburg, NJ, USA). The antioxidants, L-ascorbic acid and L-glutathione, were purchased from Sigma-Aldrich (St. Louis, MO, USA).

### 3.2. The Preparation of the Welsh Onion Samples by Heating

A total of 800 g of washed Welsh onions and 800 g of purified water were ground in a blender, and 1.5 kg of Welsh onion juice was obtained. The Welsh onion sample was placed into a 1 L flask and heated in a 2 L oil bath. After preheating at 70 °C for 5 min, it was heated at 100 °C for 10 min.

### 3.3. The Storage Conditions of the Heated Welsh Onion Samples

Each 20 mL of heated Welsh onion samples in a 50 mL amber laboratory bottle was stored at 25 °C in constant temperature and humidity chamber (Han Baek Scientific Co., Bucheon-si, Korea). The different antioxidants, L-ascorbic acid and L-glutathione, were added to the heated Welsh onion samples at a concentration of 0.05g/100g (*w*/*w*). The Welsh onion samples were sealed with a PBT screw cap and coated silicon PTFE gasket to prevent the loss of volatile compounds. During the storage periods, the samples were obtained at 0, 1, 3, 5, and 7 days.

### 3.4. The Extraction of Volatile Compounds Using SPME

A sample of Welsh onions (5 g) was placed in a 20 mL vial; β-damascone (1 mg/mLin β-damascone/methanol solvent mixture (1:200, *v*/*v*)) was added as an internal standard, and the vial was sealed with a screw cap. SPME was used to obtain the volatile compounds of Welsh onions. The sample was maintained at 60 °C for 30 min to reach a state of equilibrium. An SPME fiber coated with carboxen/polydimethylsiloxane (CAR/PDMS) was used to adsorb volatile compounds at 40 °C for 20 min, and desorption was executed at 230 °C in a GC injector for 5 min.

### 3.5. GC-MS Analysis

GC-MS analysis was performed using a 7890A series gas chromatograph (Agilent Technologies) and a 5975C mass detector (Agilent Technologies) equipped with a DB-5MS column (30 m length × 0.25 mm i.d. × 0.25 μm film thickness, J&W Scientific, Folsom, CA, USA). The GC oven temperature was programed as follows: the initial temperature was maintained at 40 °C for 6 min, raised to 170 °C at a rate of 5 °C/min and held for 3 min, and increased to 220 °C at a rate of 5 °C/min. The flow rate of helium, the carrier gas, was constant at 0.8 mL/min, whereas the mass spectra was obtained with a mass scan rage of 35–350 atomic mass units (a.m.u.) at a rate of 4.5 scans/s, and the electron impact (EI) mode was 70 eV. All the samples were prepared and analyzed in triplicate.

### 3.6. The Identification and Semi-Quantification of the Volatile Compounds

The identification of each volatile compound was positively confirmed by a comparison of the retention time and mass spectral data with those of the authentic standard compounds. When the standard compounds were not available, each volatile compound was identified on the basis of its mass spectral data using the NIST.08 and Wiley.9 mass spectral libraries and the retention index (RI) values from the previous literature [1,2,20,26,27]. The RI value of the volatile compounds was calculated with the n-alkanes from C_6_ to C_30_ as the external standards. The quantification of the volatile compounds was performed by comparing their peak areas with that of the internal standard compound on the total ion chromatogram of GC-MS.

### 3.7. The Statistical Analysis

The significance of the differences (*p* < 0.05) between the untreated Welsh onion samples and the heated Welsh onion samples were evaluated by Duncan’s *t*-test using the SPSS program (version 12.0, Chicago, IL, USA). Multivariate statistical analysis, partial least square discriminant analysis (PLS-DA), was conducted using SIMCA-P (version 11.0, Umetrics, Umea, Sweden) to determine the key volatile compounds related to the discrimination of the heated Welsh onions added with antioxidants.

## 4. Conclusions

This study investigated the changes in the volatile compounds in Welsh onions by heat treatment and the effects of antioxidants (ascorbic acid and glutathione) on the changes in the volatile compounds during storage. Some sulfur-containing compounds, such as 2-methylthiirane (propylene sulfide); 1-(methyldisulfanyl)prop-1-ene; 1-[[(E)-prop-1-enyl]disulfanyl]propane (propyl (E)-1-propenyl disulfide); 1-(propyltrisulfanyl)propane; 1-[[(E)-prop-1-enyl]trisulfanyl]propane; and (methyltetrasulfanyl)methane (dimethyl tetrasulfide), showed significant differences between the untreated Welsh onion and heated Welsh onion samples. These results demonstrate that heat treatment can affect the flavor profiles of Welsh onions, through their thermal decomposition and rearrangement.

The application of PLS-DA to the data sets of volatile compounds revealed that heated Welsh onion samples can be distinguished according to different antioxidants, such as ascorbic acid and glutathione. 3-ethylthiophene; 1-(methyldisulfanyl)-1-methylsulfanylpropane; 1-methylsulfanylpentane; 2-prop-2-enylsulfanylpropane; and 1-propan-2-ylsulfanylbutane were strongly correlated with the heated Welsh onion samples added with glutathione. On the other hand, 2-phenylacetaldehyde; acetic acid; methylsulfanylmethane; prop-2-ene-1-thiol; undecan-2-one; and (2E,4E)-deca-2,4-dienal were related to the heated Welsh onion samples added with ascorbic acid. These findings indicate that the profiles of the volatile compounds of the heated Welsh onion samples added with different antioxidants can significantly change the volatile profiles during storage. These results can be used to improve the quality of Welsh onion-based products and develop processed foods.

## Figures and Tables

**Table 1:** Volatile compounds identified in the untreated and heated Welsh onion samples.

No ^(1)^	RI ^(2)^	Volatile Compounds	Relative Peak Area ^(3)^		ID ^(4)^
			Untreated Welsh Onion	Heated Welsh Onion	
**Acids**					
c1	<600	Acetic acid	0.064 ± 0.007	0.015 ± 0.004	A
c2	685	Propanoic acid	0.002 ± 0.000	N.D. ^(6)^	A
c3	965	Heptanoic acid	0.067 ± 0.001	N.D. a ^(5)^	A
c4	1101	2-methylpentanoic acid	0.024 ± 0.003	0.014 ± 0.004	C
c5	1152	Benzoic acid	0.041 ± 0.008	0.057 ± 0.007	A
c6	1254	Nonanoic acid	0.067 ± 0.002	0.011 ± 0.002 a	A
**Alcohols**					
a1	682	pent-1-en-3-ol	0.010 ± 0.001	N.D. a	C
a2	715	1-(1-propynyl)cyclopropanol	0.175 ± 0.011	0.240 ± 0.037	C
a3	768	pentan-1-ol	0.043 ± 0.008	0.004 ± 0.001 a	A
a4	866	(E)-hex-2-en-1-ol	0.007 ± 0.001	N.D.	A
a5	869	hexan-1-ol	0.201 ± 0.010	0.015 ± 0.001	A
a6	999	2-(2-ethoxyethoxy)ethanol	N.D.	0.024 ± 0.006 a	A
a7	1029	2-ethylhexan-1-ol	0.207 ± 0.050	0.076 ± 0.018	A
a8	1049	1-ethenylcyclohexan-1-ol	0.006 ± 0.001	0.006 ± 0.002	C
a9	1060	1-phenylethane-1,2-diol	N.D.	0.064 ± 0.008 a	C
a10	1070	octan-1-ol	0.058 ± 0.003	0.040 ± 0.008	A
a11	1283	(2E)-3,7-dimethylocta-2,6-dien-1-ol	0.008 ± 0.000	N.D. a	A
a12	1502	tridecan-2-ol	0.002 ± 0.000	N.D. a	B
**Aldehydes**					
d1	<600	Acetaldehyde	0.219 ± 0.057	0.042 ± 0.005 a	A
d2	<600	Propanal	0.972 ± 0.058	0.763 ± 0.076	C
d3	699	Pentanal	0.042 ± 0.006	0.012 ± 0.002	A
d4	701	(2E,4E)-hexa-2,4-dienal	0.026 ± 0.006	N.D. a	A
d5	744	(E)-2-methylbut-2-enal	0.003 ± 0.000	0.002 ± 0.001	B
d6	757	(E)-pent-2-enal	0.078 ± 0.004	0.022 ± 0.004	A
d7	801	Hexanal	2.763 ± 0.367	0.512 ± 0.075 a	A
d8	834	(E)-2-methylpent-2-enal	18.255 ± 1.756	6.490 ± 1.003 a	A
d9	848	(E)-2-ethylbut-2-enal	1.563 ± 0.356	N.D. a	B
d10	856	(E)-hex-2-enal	0.887 ± 0.048	0.159 ± 0.026 a	A
d11	903	Heptanal	0.201 ± 0.017	0.026 ± 0.005 a	A
d12	960	(E)-hept-2-enal	0.266 ± 0.021	0.101 ± 0.007	A
d13	969	Benzaldehyde	0.107 ± 0.013	0.095 ± 0.026	A
d14	1005	Octanal	0.084 ± 0.013	0.065 ± 0.005	A
d15	1014	(2E,4E)-hepta-2,4-dienal	0.171 ± 0.006	0.044 ± 0.005 a	A
d16	1051	2-phenylacetaldehyde	0.011 ± 0.002	0.010 ± 0.001	A
d17	1061	(E)-oct-2-enal	0.307 ± 0.027	0.085 ± 0.009 a	B
d18	1106	Nonanal	0.280 ± 0.070	0.381 ± 0.042	A
d19	1157	(2E,6Z)-nona-2,6-dienal	0.019 ± 0.003	N.D. a	C
d20	1164	(E)-non-2-enal	0.045 ± 0.011	N.D. a	A
d21	1208	Decanal	0.065 ± 0.012	0.117 ± 0.015	A
d22	1221	(2E,4E)-nona-2,4-dienal	0.014 ± 0.003	N.D.	C
d23	1232	2,6,6-trimethylcyclohexene-1-carbaldehyde	0.173 ± 0.033	0.199 ± 0.026	A
d24	1322	(2E,4E)-deca-2,4-dienal	0.011 ± 0.003	0.045 ± 0.009	A
**Esters**					
e1	<600	Methyl acetate	0.053 ± 0.018	N.D. a	A
e2	625	Methyl propanoate	0.016 ± 0.006	N.D.	A
e3	1009	Propanoyl propanoate	0.016 ± 0.002	0.025 ± 0.003	C
**Furans**					
f1	701	2-ethylfuran	N.D.	0.011 ± 0.002 a	A
f2	993	2-pentylfuran	0.311 ± 0.030	0.173 ± 0.044	
**Hydrocarbons**					
h1	1001	Decane	0.127 ± 0.014	0.029 ± 0.007	A
h2	1200	Dodecane	0.031 ± 0.002	0.026 ± 0.005	A
h3	1299	Tridecane	0.009 ± 0.002	0.013 ± 0.001	A
h4	1399	Tetradecane	0.036 ± 0.008	0.094 ± 0.017	A
h5	1499	Pentadecane	0.004 ± 0.000	0.006 ± 0.001	A
**Ketones**					
k1	<600	butane-2,3-dione	0.003 ± 0.000	N.D.	A
k2	660	1-hydroxypropan-2-one	0.015 ± 0.003	0.004 ± 0.001	A
k3	1293	undecan-2-one	0.034 ± 0.011	0.297 ± 0.013 a	A
k4	1495	tridecan-2-one	0.113 ± 0.036	0.458 ± 0.035 a	A
**S-containing compounds**					
**Thiols**					
s1	610	propane-1-thiol	0.009 ± 0.003	0.009 ± 0.000	A
s2	618	prop-2-ene-1-thiol	0.009 ± 0.004	0.108 ± 0.030	A
**Sulfides**					
s3	<600	Methylsulfanylmethane	0.027 ± 0.004	0.097 ± 0.015 a	A
s4	615	2-methylthiirane	0.098 ± 0.020	0.112 ± 0.028 a	B
s5	738	3-methylsulfanylprop-1-ene	0.007 ± 0.002	0.006 ± 0.000	A
s6	923	3-prop-2-enylsulfanylprop-1-ene	0.217 ± 0.026	0.405 ± 0.053	A
**Disulfides**					
s7	747	(methyldisulfanyl)methane	0.543 ± 0.022	0.571 ± 0.090	A
s8	937	1-(methyldisulfanyl)prop-1-ene	8.714 ± 0.752	16.535 ± 2.027 a	B
s9	1090	1-(methyldisulfanyl)propan-2-one	N.D.	0.021 ± 0.002 a	C
s10	1125	1-[[(E)-prop-1-enyl]disulfanyl]propane	2.206 ± 0.396	8.201 ± 0.733 a	B
s11	1274	1-(methyldisulfanyl)-1-Methylsulfanylpropane	N.D.	0.019 ± 0.003 a	B
**Trisulfides**					
s12	981	(methyltrisulfanyl)methane	10.084 ± 1.007	12.056 ± 1.161	A
s13	1154	3-(methyltrisulfanyl)prop-1-ene	0.025 ± 0.002	N.D.	B
s14	1342	1-(propyltrisulfanyl)propane	0.066 ± 0.016	2.063 ± 0.333 a	A
s15	1356	1-[[(E)-prop-1-enyl]trisulfanyl]propane	0.716 ± 0.222	7.912 ± 1.042 a	B
s16	1363	1-[[(Z)-prop-1-enyl]trisulfanyl]propane	0.182 ± 0.041	0.461 ± 0.041	B
**Tetrasulfides**					
s17	1239	(methyltetrasulfanyl)methane	1.480 ± 0.393	3.595 ± 0.279 a	B
**Cyclic sulfur compounds**					
s18	882	2,4-dimethylthiophene	1.101 ± 0.042	1.235 ± 0.128	B
s19	892	2,3-dimethylthiophene	0.025 ± 0.001	0.037 ± 0.004	B
s20	901	1,3-dithiane	0.029 ± 0.002	0.005 ± 0.001	A
s21	909	2,5-dimethylthiophene	5.598 ± 0.146	5.337 ± 0.758	A
s22	919	3,4-dimethylthiophene	0.203 ± 0.028	0.359 ± 0.062	B
s23	1093	5-methylthiophene-2-carbaldehyde	N.D.	0.025 ± 0.005 a	A
s24	1103	3-methylthiophene-2-carbaldehyde	0.025 ± 0.002	N.D. a	A
s25	1267	3,5-dimethyl-2-(methylthio)-thiophene	0.036 ± 0.003	0.026 ± 0.008	B

^(1)^ All volatile compounds are listed by the order of their RI values in chemical class. ^(2)^ Retention indices were determined using n-alkanes C_6_–C_30_ as external standards. ^(3)^ Mean values of the relative peak area to that of the internal standard ± standard deviation (*n* = 3). ^(4)^ Identification of the compounds was based on the following: A, the mass spectrum and retention index agreed with those of the authentic compounds under the same conditions (positive identification); B, the mass spectrum and retention index were consistent with those from the NIST database (tentative identification); and C, the mass spectrum was consistent with that of W9N08 (Wiley and NIST) and manual interpretation (tentative identification). ^(5)^ The letter ‘a’ indicates a significant difference between the two samples in a row using the *t*-test (*p* < 0.05). ^(6)^ N.D. = not detected.

**Table 2:** Volatile compounds identified in the heated Welsh onion samples added with antioxidants during storage.

No ^(1)^	RI ^(2)^	Volatile Compounds	Antioxidant	Relative Peak Area ^(3)^					ID ^(5)^
				Storage Periods ^(4)^					
				0 Day	1 Day	3 Days	5 Days	7 Days	
**Acids**									
c1	<600	Acetic acid	Control	0.111 ± 0.016 c ^(6)^	0.062 ± 0.005 b	0.024 ± 0.003 a	0.033 ± 0.013 a	0.019 ± 0.007 a	A
			Ascorbic acid		0.116 ± 0.017 b	0.170 ± 0.018 c	0.111 ± 0.011 b	0.062 ± 0.013 a	
			Glutathione		0.097 ± 0.014 b	0.081 ± 0.008 b	0.083 ± 0.009 b	0.047 ± 0.003 a	
c2	1101	2-methylpentanoic acid	Control	0.035 ± 0.010 b	0.010 ± 0.001 a	0.006 ± 0.001 a	0.005 ± 0.001 a	0.003 ± 0.000 a	C
			Ascorbic acid		0.042 ± 0.006 c	0.017 ± 0.002 b	0.019 ± 0.003 b	0.007 ± 0.001 a	
			Glutathione		0.052 ± 0.002 d	0.029 ± 0.000 c	0.023 ± 0.003 b	0.013 ± 0.001 a	
**Alcohols**									
a1	768	pentan-1-ol	Control	0.003 ± 0.000 bc	0.003 ± 0.001 c	0.002 ± 0.000 ab	0.002 ± 0.000 a	0.002 ± 0.000 ab	A
			Ascorbic acid		0.003 ± 0.000 c	0.004 ± 0.000 c	0.002 ± 0.000 b	N.D. a	
			Glutathione		0.002 ± 0.000 c	0.003 ± 0.000 d	0.001 ± 0.000 a	0.002 ± 0.000 b	
a2	869	hexan-1-ol	Control	0.045 ± 0.002 d	0.023 ± 0.003 c	0.011 ± 0.001 b	0.001 ± 0.000 a	N.D. a	A
			Ascorbic acid		0.042 ± 0.002 c	0.033 ± 0.009 c	0.014 ± 0.002 b	0.003 ± 0.001 a	
			Glutathione		N.D. ^(7)^	N.D.	N.D.	N.D.	
a3	1029	2-ethylhexan-1-ol	Control	0.056 ± 0.015 c	0.028 ± 0.004 ab	0.029 ± 0.006 b	0.020 ± 0.001 ab	0.014 ± 0.002 a	A
			Ascorbic acid		0.022 ± 0.004 b	0.016 ± 0.001 a	0.016 ± 0.002 a	0.014 ± 0.002 a	
			Glutathione		0.014 ± 0.002 c	0.010 ± 0.001 b	0.009 ± 0.001 b	0.004 ± 0.001 a	
a4	1070	octan-1-ol	Control	0.030 ± 0.004 c	0.036 ± 0.002 d	0.018 ± 0.003 b	0.017 ± 0.001 b	0.009 ± 0.002 a	A
			Ascorbic acid		0.046 ± 0.006 d	0.036 ± 0.002 c	0.021 ± 0.001 b	0.013 ± 0.002 a	
			Glutathione		0.037 ± 0.004 b	0.036 ± 0.005 b	0.027 ± 0.006 a	0.022 ± 0.002 a	
a5	1502	tridecan-2-ol	Control	0.005 ± 0.001 a	0.007 ± 0.001 bc	0.006 ± 0.001 b	0.008 ± 0.001 c	0.007 ± 0.000 bc	B
			Ascorbic acid		0.003 ± 0.000 a	0.003 ± 0.001 a	0.006 ± 0.000 b	0.007 ± 0.000 c	
			Glutathione		N.D.	N.D.	N.D.	N.D.	
**Aldehydes**									
d1	<600	Propanal	Control	0.558 ± 0.057 c	0.578 ± 0.162 c	0.401 ± 0.020 b	0.367 ± 0.025 b	0.182 ± 0.073 a	B
			Ascorbic acid		0.390 ± 0.114 b	0.379 ± 0.020 b	0.208 ± 0.088 a	0.151 ± 0.012 a	
			Glutathione		0.397 ± 0.030 c	0.288 ± 0.006 b	0.283 ± 0.011 b	0.110 ± 0.004 a	
d2	699	Pentanal	Control	0.009 ± 0.001 b	0.009 ± 0.001 b	0.002 ± 0.000 a	0.003 ± 0.001 a	0.002 ± 0.000 a	A
			Ascorbic acid		0.008 ± 0.002 b	0.003 ± 0.000 a	0.003 ± 0.000 a	0.002 ± 0.000 a	
			Glutathione		0.009 ± 0.002 b	0.010 ± 0.001 b	0.010 ± 0.001 b	0.004 ± 0.000 a	
d3	757	(E)-pent-2-enal	Control	0.027 ± 0.009 d	0.016 ± 0.004 c	0.014 ± 0.002 bc	0.007 ± 0.002 ab	0.002 ± 0.001 a	A
			Ascorbic acid		0.012 ± 0.002 c	0.006 ± 0.000 b	N.D. a	N.D. a	
			Glutathione		0.005 ± 0.001 c	0.004 ± 0.000 b	N.D. a	N.D. a	
d4	801	Hexanal	Control	0.427 ± 0.004 d	0.373 ± 0.010 c	0.165 ± 0.018 b	0.146 ± 0.009 b	0.089 ± 0.008 a	A
			Ascorbic acid		0.201 ± 0.023 d	0.158 ± 0.016 c	0.048 ± 0.008 b	0.009 ± 0.002 a	
			Glutathione		0.615 ± 0.061 c	0.384 ± 0.027 b	0.358 ± 0.030 b	0.163 ± 0.021 a	
d5	834	(E)-2-methylpent-2-enal	Control	4.762 ± 0.899 c	5.557 ± 0.196 d	2.771 ± 0.102 b	2.619 ± 0.283 b	1.646 ± 0.121 a	A
			Ascorbic acid		3.946 ± 0.344 b	3.721 ± 0.036 b	2.688 ± 0.019 a	2.634 ± 0.129 a	
			Glutathione		3.341 ± 0.171 d	2.701 ± 0.129 c	2.086 ± 0.244 b	1.006 ± 0.345 a	
d6	856	(E)-hex-2-enal	Control	0.145 ± 0.005 d	0.116 ± 0.017 c	0.058 ± 0.002 b	0.051 ± 0.004 b	N.D. a	A
			Ascorbic acid		0.064 ± 0.009 d	0.050 ± 0.005 c	0.032 ± 0.005 b	N.D. a	
			Glutathione		0.028 ± 0.005 c	0.024 ± 0.003 b	0.019 ± 0.003 b	N.D. a	
d7	903	Heptanal	Control	0.019 ± 0.003 c	0.020 ± 0.001 c	0.006 ± 0.001 ab	0.008 ± 0.002 b	0.004 ± 0.000 a	A
			Ascorbic acid		0.016 ± 0.001 d	0.010 ± 0.001 c	0.007 ± 0.000 b	0.004 ± 0.001 a	
			Glutathione		0.013 ± 0.000 a	0.021 ± 0.004 b	0.019 ± 0.001 b	0.012 ± 0.001 a	
d8	960	(E)-hept-2-enal	Control	0.074 ± 0.006 c	0.056 ± 0.002 b	0.022 ± 0.001 a	0.021 ± 0.002 a	0.016 ± 0.002 a	A
			Ascorbic acid		0.110 ± 0.007 c	0.060 ± 0.007 b	0.026 ± 0.001 a	0.018 ± 0.001 a	
			Glutathione		0.076 ± 0.007 c	0.026 ± 0.004 b	0.013 ± 0.001 a	0.006 ± 0.002 a	
d9	969	Benzaldehyde	Control	0.069 ± 0.013 c	0.027 ± 0.004 b	0.026 ± 0.005 b	0.018 ± 0.001 b	0.006 ± 0.001 a	A
			Ascorbic acid		0.032 ± 0.007 a	0.028 ± 0.005 a	0.027 ± 0.014 a	0.019 ± 0.003 a	
			Glutathione		N.D.	N.D.	N.D.	N.D.	
d10	1005	Octanal	Control	0.048 ± 0.004 c	0.041 ± 0.001 b	0.009 ± 0.001 a	0.009 ± 0.001 a	0.008 ± 0.001 a	A
			Ascorbic acid		0.044 ± 0.004 d	0.025 ± 0.004 c	0.016 ± 0.002 b	0.005 ± 0.000 a	
			Glutathione		0.025 ± 0.006 b	0.014 ± 0.001 a	0.024 ± 0.002 b	0.012 ± 0.002 a	
d11	1014	(2E,4E)-hepta-2,4-dienal	Control	0.032 ± 0.002 c	0.020 ± 0.004 b	0.011 ± 0.002 a	0.011 ± 0.001 a	0.018 ± 0.005 b	A
			Ascorbic acid		0.010 ± 0.000 d	0.009 ± 0.001 c	0.008 ± 0.000 b	0.004 ± 0.000 a	
			Glutathione		N.D.	N.D.	N.D.	N.D.	
d12	1051	2-phenylacetaldehyde	Control	0.008 ± 0.000 b	0.007 ± 0.001 b	0.005 ± 0.000 a	0.005 ± 0.000 a	0.004 ± 0.001 a	A
			Ascorbic acid		0.020 ± 0.003 b	0.021 ± 0.003 b	0.018 ± 0.002 b	0.009 ± 0.001 a	
			Glutathione		0.009 ± 0.001 d	0.007 ± 0.001 c	0.005 ± 0.001 b	N.D. a	
d13	1061	(E)-oct-2-enal	Control	0.062 ± 0.005 b	0.057 ± 0.012 b	0.030 ± 0.004 a	0.030 ± 0.006 a	0.021 ± 0.004 a	B
			Ascorbic acid		0.054 ± 0.003 c	0.037 ± 0.003 b	0.034 ± 0.003 b	0.021 ± 0.002 a	
			Glutathione		0.041 ± 0.011 b	0.015 ± 0.002 a	0.016 ± 0.004 a	0.014 ± 0.002 a	
d14	1106	Nonanal	Control	0.278 ± 0.015 c	0.240 ± 0.019 c	0.122 ± 0.001 b	0.113 ± 0.020 b	0.068 ± 0.007 a	A
			Ascorbic acid		0.206 ± 0.010 d	0.141 ± 0.004 c	0.079 ± 0.015 b	0.027 ± 0.005 a	
			Glutathione		0.214 ± 0.004 d	0.164 ± 0.005 c	0.139 ± 0.016 b	0.085 ± 0.006 a	
d15	1208	Decanal	Control	0.085 ± 0.004 c	0.087 ± 0.009 c	0.061 ± 0.006 b	0.051 ± 0.008 b	0.036 ± 0.005 a	A
			Ascorbic acid		0.085 ± 0.003 d	0.054 ± 0.003 c	0.039 ± 0.002 b	0.029 ± 0.000 a	
			Glutathione		0.054 ± 0.001 b	0.045 ± 0.003 a	0.044 ± 0.006 a	0.043 ± 0.006 a	
d16	1322	(2E,4E)-deca-2,4-dienal	Control	0.033 ± 0.000 a	0.028 ± 0.004 a	0.030 ± 0.003 a	0.033 ± 0.003 a	0.031 ± 0.003 a	A
			Ascorbic acid		0.041 ± 0.006 b	0.043 ± 0.003 b	0.036 ± 0.005 ab	0.031 ± 0.002 a	
			Glutathione		N.D.	N.D.	N.D.	N.D.	
**Esters**									
e1	1009	Propanoyl propanoate	Control	0.018 ± 0.001 e	0.016 ± 0.001 d	0.010 ± 0.001 c	0.009 ± 0.001 b	0.006 ± 0.001 a	C
			Ascorbic acid		0.029 ± 0.003 c	0.019 ± 0.003 b	0.019 ± 0.001 b	0.008 ± 0.001 a	
			Glutathione		0.012 ± 0.001 b	0.006 ± 0.001 ab	0.004 ± 0.000 a	0.003 ± 0.000 a	
**Furans**									
f1	701	2-ethylfuran	Control	0.016 ± 0.002 d	0.008 ± 0.001 c	0.005 ± 0.000 b	0.004 ± 0.000 b	0.002 ± 0.000 a	A
			Ascorbic acid		0.018 ± 0.007 b	0.009 ± 0.001 a	0.008 ± 0.001 a	0.004 ± 0.001 a	
			Glutathione		0.012 ± 0.002 b	0.010 ± 0.004 ab	0.009 ± 0.002 ab	0.005 ± 0.001 a	
f2	993	2-pentylfuran	Control	0.133 ± 0.020 d	0.122 ± 0.006 d	0.097 ± 0.003 c	0.064 ± 0.005 b	0.033 ± 0.003 a	C
			Ascorbic acid		0.107 ± 0.021 b	0.107 ± 0.004 b	0.063 ± 0.011 a	0.041 ± 0.003 a	
			Glutathione		0.121 ± 0.005 c	0.150 ± 0.017 d	0.088 ± 0.013 b	0.046 ± 0.003 a	
**Hydrocarbons**									
h1	1000	Decane	Control	0.021 ± 0.002 b	0.014 ± 0.001 a	0.014 ± 0.002 a	0.014 ± 0.001 a	0.012 ± 0.001 a	A
			Ascorbic acid		0.013 ± 0.001 b	0.006 ± 0.001 a	0.004 ± 0.000 a	0.004 ± 0.000 a	
			Glutathione		0.017 ± 0.001 b	0.014 ± 0.001 a	0.013 ± 0.002 a	0.014 ± 0.001 a	
h2	1200	Dodecane	Control	0.019 ± 0.004 ab	0.020 ± 0.002 b	0.017 ± 0.001 ab	0.018 ± 0.001 ab	0.015 ± 0.000 a	A
			Ascorbic acid		0.013 ± 0.003 b	0.008 ± 0.001 a	0.009 ± 0.001 a	0.008 ± 0.000 a	
			Glutathione		0.017 ± 0.002 a	0.014 ± 0.001 a	0.014 ± 0.002 a	0.014 ± 0.001 a	
h3	1300	Tridecane	Control	0.010 ± 0.000 c	0.007 ± 0.001 b	0.004 ± 0.001 a	0.004 ± 0.000 a	0.003 ± 0.000 a	C
			Ascorbic acid		0.005 ± 0.000 c	0.004 ± 0.001 b	0.004 ± 0.000 ab	0.003 ± 0.000 a	
			Glutathione		0.007 ± 0.000 a	0.007 ± 0.000 a	0.007 ± 0.000 a	0.006 ± 0.000 a	
h4	1400	Tetradecane	Control	0.069 ± 0.002 b	0.035 ± 0.010 a	0.043 ± 0.005 a	0.043 ± 0.004 a	0.036 ± 0.004 a	A
			Ascorbic acid		0.045 ± 0.002 b	0.050 ± 0.004 c	0.036 ± 0.003 a	0.037 ± 0.001 a	
			Glutathione		0.014 ± 0.002 a	0.022 ± 0.001 b	0.023 ± 0.003 b	0.034 ± 0.003 c	
h5	1500	Pentadecane	Control	0.008 ± 0.002 b	0.007 ± 0.000 b	0.007 ± 0.001 b	0.007 ± 0.000 b	0.004 ± 0.001 a	C
			Ascorbic acid		0.009 ± 0.001 b	0.008 ± 0.001 b	0.008 ± 0.000 b	0.006 ± 0.000 a	
			Glutathione		N.D.	N.D.	N.D.	N.D.	
**Ketones**									
k1	660	1-hydroxypropan-2-one	Control	0.077 ± 0.012 d	0.045 ± 0.005 c	0.029 ± 0.005 b	N.D. a	N.D. a	A
			Ascorbic acid		0.071 ± 0.009 d	0.045 ± 0.004 c	0.019 ± 0.008 b	0.006 ± 0.002 a	
			Glutathione		0.074 ± 0.016 c	0.047 ± 0.008 b	0.031 ± 0.008 b	0.011 ± 0.003 a	
k2	1293	undecan-2-one	Control	0.219 ± 0.027 a	0.298 ± 0.102 a	0.498 ± 0.043 b	0.577 ± 0.055 b	0.473 ± 0.058 b	B
			Ascorbic acid		0.604 ± 0.085 ab	0.719 ± 0.047 c	0.669 ± 0.057 bc	0.499 ± 0.011 a	
			Glutathione		0.675 ± 0.106 a	0.712 ± 0.064 a	0.722 ± 0.084 a	0.705 ± 0.044 a	
k3	1495	tridecan-2-one	Control	0.335 ± 0.021 a	0.245 ± 0.056 a	0.553 ± 0.116 b	0.617 ± 0.088 b	0.821 ± 0.179 c	A
			Ascorbic acid		0.433 ± 0.024 a	0.770 ± 0.146 b	0.805 ± 0.126 b	0.746 ± 0.027 b	
			Glutathione		0.390 ± 0.082 a	0.554 ± 0.099 b	0.604 ± 0.084 b	0.879 ± 0.021 c	
**S-containing compounds**									
**Thiols**									
s1	<600	Methanethiol	Control	0.007 ± 0.001 a	0.012 ± 0.001 b	0.017 ± 0.003 c	0.026 ± 0.001 d	0.005 ± 0.001 a	C
			Ascorbic acid		0.012 ± 0.002 a	0.015 ± 0.001 a	0.015 ± 0.001 a	0.027 ± 0.002 b	
			Glutathione		0.016 ± 0.002 a	0.211 ± 0.069 b	0.279 ± 0.059 b	0.220 ± 0.034 b	
s2	610	propane-1-thiol	Control	0.006 ± 0.000 c	0.009 ± 0.001 d	0.007 ± 0.000 c	0.005 ± 0.000 b	0.003 ± 0.000 a	A
			Ascorbic acid		0.010 ± 0.001 a	0.049 ± 0.004 b	0.173 ± 0.033 c	0.154 ± 0.008 c	
			Glutathione		0.041 ± 0.004 a	0.368 ± 0.017 c	0.367 ± 0.031 c	0.231 ± 0.026 b	
s3	618	prop-2-ene-1-thiol	Control	0.091 ± 0.019 ab	0.081 ± 0.038 a	0.159 ± 0.051 b	0.072 ± 0.052 a	0.057 ± 0.020 a	A
			Ascorbic acid		0.235 ± 0.030 a	0.272 ± 0.111 a	0.543 ± 0.206 b	0.128 ± 0.020 a	
			Glutathione		0.357 ± 0.024 c	0.306 ± 0.026 b	0.161 ± 0.016 a	0.157 ± 0.021 a	
**Sulfides**									
s4	<600	methylsulfanylmethane(dimethyl sulfide)	Control	0.071 ± 0.006 c	0.074 ± 0.014 c	0.054 ± 0.007 b	0.040 ± 0.006 b	0.020 ± 0.004 a	A
			Ascorbic acid		0.096 ± 0.005 b	0.092 ± 0.003 b	0.117 ± 0.010 c	0.055 ± 0.012 a	
			Glutathione		0.072 ± 0.008 d	0.050 ± 0.005 c	0.030 ± 0.002 b	0.018 ± 0.008 a	
s5	615	2-methylthiirane(propylene sulfide)	Control	0.082 ± 0.011 c	0.022 ± 0.001 a	0.030 ± 0.003 ab	0.051 ± 0.008 b	0.038 ± 0.021 ab	B
			Ascorbic acid		0.120 ± 0.025 ab	0.176 ± 0.019 b	0.316 ± 0.062 c	0.078 ± 0.004 a	
			Glutathione		0.048 ± 0.002 a	0.275 ± 0.041 d	0.181 ± 0.016 c	0.131 ± 0.008 b	
s6	738	3-methylsulfanylprop-1-ene(allyl methyl sulfide)	Control	0.005 ± 0.000 a	0.008 ± 0.001 b	0.007 ± 0.001 b	0.005 ± 0.000 a	0.005 ± 0.001 a	A
			Ascorbic acid		0.008 ± 0.001 b	0.005 ± 0.001 a	0.004 ± 0.000 a	0.005 ± 0.000 a	
			Glutathione		0.002 ± 0.000 a	0.014 ± 0.001 c	0.018 ± 0.001 d	0.008 ± 0.002 b	
s7	920	1-methylsulfanylpentane(amyl methyl sulfide)	Control	N.D.	N.D.	N.D.	N.D.	N.D.	C
			Ascorbic acid		N.D.	N.D.	N.D.	N.D.	
			Glutathione		0.121 ± 0.026 b	0.097 ± 0.037 ab	0.091 ± 0.010 ab	0.066 ± 0.003 a	
s8	923	3-prop-2-enylsulfanylprop-1-ene(diallyl sulfide)	Control	0.295 ± 0.030 c	0.406 ± 0.034 d	0.144 ± 0.012 b	0.091 ± 0.016 a	0.057 ± 0.009 a	A
			Ascorbic acid		0.158 ± 0.031 b	0.176 ± 0.019 b	0.153 ± 0.018 b	0.057 ± 0.006 a	
			Glutathione		0.174 ± 0.018 c	0.125 ± 0.006 b	0.094 ± 0.013 a	0.085 ± 0.004 a	
s9	1023	1-propan-2-ylsulfanylbutane(butyl isopropyl sulfide)	Control	N.D.	N.D.	N.D.	N.D.	N.D.	C
			Ascorbic acid		N.D.	N.D.	N.D.	N.D.	
			Glutathione		0.019 ± 0.003 c	0.016 ± 0.001 b	0.007 ± 0.001 a	0.006 ± 0.001 a	
s10	1450	2-prop-2-enylsulfanylpropane(allyl isopropyl sulfide)	Control	0.064 ± 0.008 a	0.053 ± 0.005 a	0.246 ± 0.042 b	0.245 ± 0.033 b	0.276 ± 0.012 b	C
			Ascorbic acid		0.443 ± 0.021 c	0.381 ± 0.043 b	0.343 ± 0.038 b	0.248 ± 0.007 a	
			Glutathione		0.544 ± 0.032 a	0.659 ± 0.129 a	0.648 ± 0.110 a	0.753 ± 0.130 a	
**Disulfides**									
s11	747	(methyldisulfanyl)methane(dimethyl disulfide)	Control	0.418 ± 0.067 c	0.626 ± 0.039 d	0.207 ± 0.006 b	0.170 ± 0.004 b	0.083 ± 0.010 a	A
			Ascorbic acid		0.428 ± 0.078 c	0.233 ± 0.009 b	0.251 ± 0.022 b	0.092 ± 0.012 a	
			Glutathione		0.296 ± 0.028 c	0.216 ± 0.015 b	0.233 ± 0.027 b	0.105 ± 0.017 a	
s12	937	1-(methyldisulfanyl)prop-1-ene(methyl (E)-1-propenyl disulfide)	Control	12.085 ± 1.401 c	16.625 ± 0.089 d	8.746 ± 0.392 b	7.631 ± 0.407 ab	7.141 ± 0.915 a	B
			Ascorbic acid		14.426 ± 1.186 c	9.355 ± 0.324 b	8.331 ± 0.182 b	5.022 ± 0.338 a	
			Glutathione		2.685 ± 0.230 d	1.432 ± 0.015 c	1.163 ± 0.089 b	0.560 ± 0.019 a	
s13	1125	1-[(E)-prop-1-enyl]disulfanyl]propane((E)-propenyl propyl disulfide)	Control	6.005 ± 0.642 c	7.670 ± 0.551 d	4.901 ± 0.259 b	4.422 ± 0.351 b	3.161 ± 0.294 a	B
			Ascorbic acid		7.349 ± 0.389 c	5.427 ± 0.132 b	5.435 ± 0.276 b	3.276 ± 0.118 a	
			Glutathione		7.565 ± 0.101 d	5.499 ± 0.349 c	4.530 ± 0.372 b	2.609 ± 0.117 a	
s14	1274	1-(methyldisulfanyl)-1-methylsulfanylpropane(methyl 1-(methylthio)propyl disulfide)	Control	0.014 ± 0.002 b	0.017 ± 0.002 c	0.010 ± 0.001 a	0.010 ± 0.001 a	0.009 ± 0.000 a	B
			Ascorbic acid		0.016 ± 0.001 b	0.015 ± 0.001 b	0.020 ± 0.004 c	0.010 ± 0.001 a	
			Glutathione		0.057 ± 0.002 a	0.080 ± 0.005 b	0.110 ± 0.013 c	0.083 ± 0.008 b	
s15	1318	1-(propyldisulfanyl)pentane(propyl pentyl disulfide)	Control	0.026 ± 0.004 a	0.023 ± 0.007 a	0.024 ± 0.003 a	0.025 ± 0.004 a	0.022 ± 0.001 a	C
			Ascorbic acid		0.037 ± 0.004 ab	0.036 ± 0.002 ab	0.039 ± 0.001 b	0.032 ± 0.003 a	
			Glutathione		0.031 ± 0.004 a	0.030 ± 0.003 a	0.029 ± 0.003 a	0.029 ± 0.002 a	
s16	1515	1-methyl-2-(3,5-dimethylthien-4-yl)disulfide	Control	0.055 ± 0.001 ab	0.044 ± 0.004 a	0.054 ± 0.008 ab	0.063 ± 0.011 bc	0.075 ± 0.012 c	C
			Ascorbic acid		0.045 ± 0.006 a	0.073 ± 0.008 b	0.075 ± 0.005 b	0.085 ± 0.003 c	
			Glutathione		0.012 ± 0.001 a	0.026 ± 0.001 b	0.031 ± 0.002 b	0.046 ± 0.009 c	
**Trisulfides**									
s17	981	(methyltrisulfanyl)methane(dimethyl trisulfide)	Control	8.816 ± 0.840 c	11.564 ± 0.047 d	6.360 ± 0.316 b	5.798 ± 0.282 b	3.866 ± 0.213 a	A
			Ascorbic acid		10.387 ± 0.964 d	6.942 ± 0.300 c	5.288 ± 0.219 b	4.187 ± 0.200 a	
			Glutathione		1.266 ± 0.131 c	0.840 ± 0.082 b	0.832 ± 0.167 b	0.358 ± 0.015 a	
s18	1342	1-(propyltrisulfanyl)propane(dipropyl trisulfide)	Control	1.725 ± 0.157 b	1.643 ± 0.140 b	1.575 ± 0.106 b	1.503 ± 0.182 ab	1.254 ± 0.130 a	A
			Ascorbic acid		1.779 ± 0.150 b	1.814 ± 0.097 b	1.859 ± 0.038 b	1.434 ± 0.033 a	
			Glutathione		1.087 ± 0.062 c	1.105 ± 0.119 c	0.830 ± 0.101 b	0.624 ± 0.032 a	
s19	1356	1-[(E)-prop-1-enyl]trisulfanyl]propane[propyl (E)-1-propenyl trisulfide]	Control	5.767 ± 0.500 c	5.358 ± 0.330 bc	5.248 ± 0.553 abc	4.703 ± 0.361 ab	4.557 ± 0.188 a	B
			Ascorbic acid		5.747 ± 0.454 a	5.882 ± 0.032 a	5.533 ± 0.122 a	5.641 ± 0.111 a	
			Glutathione		3.363 ± 0.016 b	3.422 ± 0.138 b	3.387 ± 0.281 b	2.793 ± 0.175 a	
s20	1363	1-[[(Z)-prop-1-enyl]trisulfanyl]propane[propyl (Z)-1-propenyl trisulfide]	Control	0.336 ± 0.007 a	0.446 ± 0.071 a	2.904 ± 0.388 b	2.876 ± 0.353 b	3.199 ± 0.454 b	B
			Ascorbic acid		0.565 ± 0.062 a	1.753 ± 0.301 b	3.446 ± 0.287 c	4.003 ± 0.501 c	
			Glutathione		3.134 ± 0.186 ab	3.271 ± 0.156 ab	3.439 ± 0.301 b	2.953 ± 0.167 a	
**Tetrasulfide**									
s21	1239	(methyltetrasulfanyl)methane(dimethyl tetrasulfide)	Control	2.625 ± 0.057 b	3.082 ± 0.134 c	2.681 ± 0.149 b	2.704 ± 0.167 b	2.343 ± 0.181 a	B
			Ascorbic acid		2.557 ± 0.199 b	2.371 ± 0.071 b	2.109 ± 0.043 a	2.561 ± 0.061 b	
			Glutathione		0.340 ± 0.028 c	0.243 ± 0.051 b	0.214 ± 0.064 b	0.100 ± 0.021 a	
**Cyclic sulfur compounds**									
s22	870	3-ethylthiophene	Control	N.D.	N.D.	N.D.	N.D.	N.D.	A
			Ascorbic acid		N.D.	N.D.	N.D.	N.D.	
			Glutathione		0.008 ± 0.001 a	0.011 ± 0.001 ab	0.013 ± 0.003 bc	0.010 ± 0.001 c	
s23	882	2,4-dimethylthiophene	Control	0.900 ± 0.016 d	1.284 ± 0.079 e	0.543 ± 0.047 c	0.427 ± 0.021 b	0.246 ± 0.022 a	B
			Ascorbic acid		1.088 ± 0.141 c	0.627 ± 0.055 b	0.483 ± 0.025 b	0.250 ± 0.016 a	
			Glutathione		1.498 ± 0.080 c	0.964 ± 0.049 b	0.999 ± 0.042 b	0.512 ± 0.023 a	
s24	892	2,3-dimethylthiophene	Control	0.027 ± 0.000 c	0.038 ± 0.003 d	0.022 ± 0.001 b	0.022 ± 0.001 b	0.009 ± 0.001 a	B
			Ascorbic acid		0.035 ± 0.002 d	0.028 ± 0.003 c	0.022 ± 0.002 b	0.013 ± 0.002 a	
			Glutathione		0.021 ± 0.002 a	0.023 ± 0.001 a	0.030 ± 0.003 b	0.031 ± 0.003 b	
s25	901	1,3-dithiane	Control	0.003 ± 0.001 d	0.003 ± 0.000 c	0.002 ± 0.000 b	N.D. a	N.D. a	A
			Ascorbic acid		0.012 ± 0.003 a	0.030 ± 0.009 b	0.029 ± 0.008 b	0.004 ± 0.002 a	
			Glutathione		0.056 ± 0.002 b	0.052 ± 0.016 b	0.045 ± 0.007 b	0.026 ± 0.004 a	
s26	909	2,5-dimethylthiophene	Control	3.902 ± 0.552 c	5.450 ± 0.349 d	2.177 ± 0.258 b	1.834 ± 0.110 b	1.114 ± 0.130 a	A
			Ascorbic acid		4.608 ± 0.672 c	5.038 ± 0.506 c	2.900 ± 0.286 b	1.206 ± 0.062 a	
			Glutathione		4.810 ± 0.408 c	4.116 ± 0.088 b	4.049 ± 0.254 b	3.098 ± 0.095 a	
s27	919	3,4-dimethylthiophene	Control	0.261 ± 0.028 c	0.263 ± 0.026 c	0.141 ± 0.005 b	0.085 ± 0.008 a	0.064 ± 0.010 a	B
			Ascorbic acid		0.411 ± 0.042 d	0.304 ± 0.017 c	0.235 ± 0.014 b	0.094 ± 0.009 a	
			Glutathione		0.161 ± 0.017 d	0.129 ± 0.011 c	0.105 ± 0.003 b	0.065 ± 0.005 a	
s28	1093	5-methylthiophene-2-carbaldehyde	Control	0.018 ± 0.001 c	0.011 ± 0.002 b	0.004 ± 0.001 a	0.003 ± 0.000 a	0.003 ± 0.000 a	A
			Ascorbic acid		0.008 ± 0.001 c	0.007 ± 0.002 bc	0.005 ± 0.001 ab	0.004 ± 0.000 a	
			Glutathione		0.008 ± 0.001 c	0.005 ± 0.000 b	0.004 ± 0.001 a	0.003 ± 0.000 b	
s29	1267	3,5-dimethyl-2-(methylthio)-thiophene	Control	0.019 ± 0.005 a	0.027 ± 0.003 b	0.025 ± 0.004 b	0.029 ± 0.001 b	0.023 ± 0.000 ab	B
			Ascorbic acid		0.034 ± 0.005 b	0.034 ± 0.002 b	0.052 ± 0.005 c	0.020 ± 0.002 a	
			Glutathione		0.015 ± 0.001 a	0.042 ± 0.006 b	0.149 ± 0.030 a	0.400 ± 0.032 c	
**Others**									
s30	701	*S*-methyl ethanethioate	Control	0.045 ± 0.004 b	0.050 ± 0.007 b	0.041 ± 0.005 b	0.041 ± 0.006 b	0.019 ± 0.006 a	A
			Ascorbic acid		0.083 ± 0.015 c	0.070 ± 0.004 bc	0.059 ± 0.007 b	0.038 ± 0.008 a	
			Glutathione		0.077 ± 0.012 c	0.065 ± 0.007 bc	0.050 ± 0.009 ab	0.045 ± 0.003 a	
s31	724	(Z)-1-methylsulfanylprop-1-ene	Control	0.004 ± 0.000 c	0.004 ± 0.000 c	0.002 ± 0.000 b	0.002 ± 0.000 b	N.D. a	B
			Ascorbic acid		0.010 ± 0.000 b	0.008 ± 0.001 a	0.008 ± 0.001 a	0.006 ± 0.001 a	
			Glutathione		0.017 ± 0.002 c	0.010 ± 0.001 b	0.012 ± 0.001 b	0.06 ± 0.001 a	

^(1)^ All volatile compounds were grouped in chemical classes and listed in the order of their RI values. (^2)^ Retention indices were determined using n-alkanes C_7_–C_30_ as external standards. ^(3)^ Mean values of the relative peak area to that of the internal standard ± standard deviation. ^(4)^ 0: unstored Welsh onion samples; 1: sample after one day; 3: sample after three days; 5: sample after five days; 7: sample after seven days; Control: heat-treatment sample; Ascorbic acid: heat-treatment sample with ascorbic acid; and Glutathione: heat-treatment sample with glutathione. ^(5)^ The identification of the compounds was based on the following: A, the mass spectrum and retention index agreed with those of the W9N08 mass spectral database, and the literatures and authentic standards; B, the mass spectrum was identical with that of the W9N08 mass spectral database, and the retention index was consistent with that in the literature; and C, the mass spectrum was consistent with that of W9N08 (Wiley and NIST) and manual interpretation. ^(6)^ Significant differences (*p* < 0.05) between the heated welsh onion samples according to the storage periods using Duncan’s multiple comparison test. ^(7)^ N.D. = not detected.

**Table 3:** The major volatile compounds identified in the heated Welsh onion samples added with different antioxidants, based on the variable importance plot (VIP > 1.0) list for PLS component 1 of PLS-DA.

Volatile Compounds	VIP Value
Positive Direction	
(methyltetrasulfanyl)methane	1.65
(2E,4E)-deca-2,4-dienal	1.56
Pentadecane	1.54
1-[[(E)-prop-1-enyl]trisulfanyl]propane	1.46
1-(propyltrisulfanyl)propane	1.36
(methyltrisulfanyl)methane	1.35
1-(methyldisulfanyl)prop-1-ene	1.34
(2E,4E)-hepta-2,4-dienal	1.23
1-methyl-2-(3,5-dimethylthien-4-yl)disulfide	1.19
Tetradecane	1.17
Benzaldehyde	1.13
**Negative Direction**	
3-ethylthiophene	1.62
1-(methyldisulfanyl)-1-methylsulfanylpropane	1.58
1-methylsulfanylpentane	1.57
2-prop-2-enylsulfanylpropane	1.45
1-propan-2-ylsulfanylbutane	1.42
1,3-dithiane	1.40
Methanethiol	1.34
1-(propyldisulfanyl)propane	1.27
propane-1-thiol	1.25
(Z)-1-methylsulfanylprop-1-ene	1.18
undecan-2-one	1.02

**Table 4:** The major volatile compounds identified in the heated Welsh onion samples added with different antioxidants, based on the variable importance plot (VIP > 1.0) list for PLS component 2 of PLS-DA.

**Volatile Compounds**	**VIP Value**
**Positive Direction**	
2-phenylacetaldehyde	1.75
Acetic acid	1.41
Methylsulfanylmethane	1.34
prop-2-ene-1-thiol	1.33
undecan-2-one	1.28
(2E,4E)-deca-2,4-dienal	1.26
1-[[(E)-prop-1-enyl]trisulfanyl]propane	1.24
2-prop-2-enylsulfanylpropane	1.19
1,3-dithiane	1.18
1-(propyltrisulfanyl)propane	1.14
1-methyl-2-(3,5-dimethylthien-4-yl)disulfide	1.06
3,4-dimethylthiophene	1.05
propane-1-thiol	1.02
**Negative Direction**	
Decane	1.65
Dodecane	1.47
(2E,4E)-hepta-2,4-dienal	1.29
(methyltetrasulfanyl)methane	1.19
3-ethylthiophene	1.18
1-methylsulfanylpentane	1.15
1-(methyldisulfanyl)-1-methylsulfanylpropane	1.14
1-propan-2-ylsulfanylbutane	1.04
Hexanal	1.03
2-ethylhexan-1-ol	1.02

## Data Availability

Not applicable.
